# Beyond *EML4*: efficacy of targeted therapy in lung cancer patients with *rare ALK* fusions – a real-world retrospective analysis

**DOI:** 10.1038/s41698-026-01486-y

**Published:** 2026-06-29

**Authors:** Marie-Elisabeth Leßmann, Felix Carl Saalfeld, Lea Ruge, Diego Kauffmann-Guerrero, Oliver Illini, Albrecht Stenzinger, Kaija Minuth-Fuchs, Achim Rittmeyer, Isabell Goetting, Katharina Schildknecht, Bastian Eul, Christoph Schubart, Sacha I. Rothschild, Christian Grohé, Karin Armster, Katja Mohorčič, Urska Janzic, Cornelius F. Waller, Tobias Raphael Overbeck, Rita Vesce, Hanna Schulte, Laetitia Arja Mauti, Susann Stephan-Falkenau, Marcel Wiesweg, Martin Faehling, Uwe Gerstenmaier, Sabine Schmid, Waleed Kian, Rostyslav Lozynskyy, Marija Ivanović, Konstantinos Syrigos, Ronald Simon, Sabine Merkelbach-Bruse, Carina Wenzel, Sascha Brückmann, Sylvia Herold, Daniela E. Aust, Michael Thomas, Maximilian Johannes Hochmair, Amanda Tufman, Anna Rasokat, National Network Genomic Medicine Lung Cancer (nNGM), Petros Christopoulos, Martin Wermke

**Affiliations:** 1 Department of Medicine I, University Hospital Carl Gustav Carus Dresden, TU Dresden, Dresden, Germany; 2 Institute for Pathology, University Hospital Carl Gustav Carus Dresden, TU Dresden, Dresden, Germany; 3 National Center for Tumor Diseases (NCT), NCT/UCC Dresden, a partnership between DKFZ, Faculty of Medicine and University Hospital Carl Gustav Carus, TUD Dresden University of Technology, and Helmholtz-Zentrum Dresden-Rossendorf (HZDR), Dresden, Germany; 4 National Network Genomic Medicine Lung Cancer (nNGM), Köln, Germany; 5 Clinic for Internal Medicine I, University Hospital, University of Cologne, Köln, Germany; 6 Department of Medicine V, University Hospital, LMU Munich, German Center for Lung Research (DZL), Munich, Germany; 7 Department of Respiratory and Critical Care Medicine, Klinik Floridsdorf, Vienna Healthcare Group, Vienna, Austria; 8 Karl Landsteiner Institute of Lung Research and Pulmonary Oncology, Vienna, Austria; 9 Institute of Pathology, Heidelberg University Hospital, Heidelberg, Germany; 10 Translational Lung Research Center Heidelberg (TLRC-H), German Center for Lung Research (DZL), Heidelberg, Germany; 11 Department of Pneumology, University Hospital Regensburg, Regensburg, Germany; 12 LKI Lungenfachklinik Immenhausen, Immenhausen, Germany; 13 Institute of Pathology and Neuropathology, University Hospital Tübingen, Tübingen, Germany; 14 Robert Bosch Krankenhaus, Department of Clinical Pathology, Stuttgart, Germany; 15 Dr. Margarete Fischer Bosch Institute of Clinical Pharmacology, Stuttgart, Germany; 16 University of Tuebingen, Tuebingen, Germany; 17 Department of Internal Medicine, Justus-Liebig-University Giessen, Universities of Giessen and Marburg Lung Center (UGMLC), Giessen, Germany; 18 Institute of Pathology, Universitätsklinikum Erlangen, Friedrich-Alexander-Universität Erlangen-Nürnberg (FAU), Erlangen, Germany; 19 Cantonal Hospital Baden, Center Oncology/Hematology, Department Internal Medicine, Baden, Switzerland and University of Basel, Basel, Switzerland; 20 Department of Respiratory Diseases, Evangelische Lungenklinik, Berlin, Germany; 21 Karl Landsteiner University of Health Sciences, Krems, Austria; 22 Division of Pneumology, University Hospital Krems, Krems, Austria; 23 Medical Oncology Unit, University Clinic Golnik, Golnik, Slovenia; 24 Medical Faculty, University of Ljubljana, Ljubljana, Slovenia; 25 Faculty of Medicine, Department of Medicine I, Hematology, Oncology and Stem Cell Transplantation, Medical Center, University of Freiburg, Freiburg, Germany; 26 Department of Hematology and Medical Oncology, University Medical Center Göttingen, Göttingen University, Göttingen, Germany; 27 Institute for Pathology, Düsseldorf University Hospital, Heinrich Heine University, Düsseldorf-Stadtbezirk, Germany; 28 Department of Medicine, Hematology/Oncology, University Hospital, University of Frankfurt, Frankfurt am Main, Germany; 29 Department of Oncology, Cantonal Hospital Winterthur, Winterthur, Switzerland; 30 Department of Pathology, Helios Klinikum Emil von Behring, Berlin, Germany; 31 West German Cancer Center, Department of Medical Oncology, University Duisburg-Essen, Essen, Germany; 32 Klinik für Kardiologie, Angiologie und Pneumologie, Klinikum Esslingen, Esslingen, Germany; 33 Institute of Pathology, University Ulm, Ulm, Germany; 34 Department of Medical Oncology, Inselspital, University Hospital Bern, Bern, Switzerland; 35 Institute of Oncology, Assuta Ashdod University Hospital, Ashdod, Israel; 36 The Hemsely Cancer Center, Shaare Zedek Medical Center, Jerusalem, Israel; 37 Division of Personalized Medical Oncology (A420), German Cancer Research Center (DKFZ), German Center for Lung Research (DZL), Heidelberg, Germany; 38 DKFZ-Hector Cancer Institute at the University Medical Center Mannheim, Medical Faculty Mannheim, University of Heidelberg, Mannheim, Germany; 39 Department of Personalized Oncology, University Hospital Mannheim, Medical Faculty Mannheim, University of Heidelberg, Mannheim, Germany; 40 Department of Oncology, University Medical Centre Maribor, Maribor, Slovenia; 41 Third Department of Internal Medicine, Sotiria Hospital, National and Kapodistrian University of Athens, Athens, Greece; 42 Institute of Pathology, University Medical Center Hamburg-Eppendorf, Hamburg, Germany; 43 Institute of Pathology, University Hospital Cologne, Cologne, Germany; 44 Thoraxklinik-Heidelberg University Hospital, Heidelberg, Germany

**Keywords:** Cancer, Oncology

## Abstract

Evidence on prognosis and optimal treatment for patients with advanced NSCLC harboring non-EML4-ALK fusions (*rare ALK*) remains limited. In a retrospective real-world cohort from 29 centers across six countries, overall survival (OS) appeared shorter in patients *with rare ALK* fusions (*n* = 51) compared with those with EML4-ALK fusions (*n* = 277; median 27 vs. 57 months, *p* = 0.08). Among patients receiving first-line therapy, those with *rare ALK* fusions experienced significantly shorter progression-free survival (PFS) with platinum-based chemotherapy than with a TKI (5 vs. 23 months; HR 3.1, 95% CI 1.2–8, *p* = 0.02). In contrast, for patients treated first-line with an ALK-TKI, ORR (85% vs. 74%; *p* = 0.9) and PFS (median 25 vs. 23 months; HR 0.9, 95% CI 0.6–1.5) were similar between *rare ALK* and EML4-ALK groups. These findings support TKIs as preferred first-line therapy for advanced NSCLC with *rare ALK* fusions.

## Introduction

The incidence of *Anaplastic Lymphoma Kinase (ALK)* fusions in non-small cell lung cancer (ALK + NSCLC) ranges between 3 and 5%. Affected patients are predominantly non-smokers and considerably younger than the general NSCLC population^1,2^. It has been clearly shown that treatment with an ALK-specific tyrosine kinase inhibitor (TKI) is superior in terms of PFS and OS compared to platinum-based combination therapy in these patients^3^. More recently, first-line treatment with the 3rd generation TKI lorlatinib has been associated with an unprecedented 5-year progression-free survival (PFS) rate of 60%^4^.

*Echinoderm Microtubule Associated Protein-Like 4 (EML4)* is the ALK fusion partner in 80%–90% of ALK + NSCLC^5^. In the remaining 10%–20%, ALK fusions involve a heterogeneous group of over 90 different partner genes (*rare ALK)*^6^. Since ALK fusions have long been assessed by break-apart FISH (fluorescence in situ hybridization) or immunohistochemistry (IHC), details about the fusion partner remained obscure until the broad adoption of sequencing tests for fusion genes in clinical practice^7^. Evidence regarding the optimal treatment of patients with these rare ALK fusions is currently limited to case reports and small case series^8^. Crizotinib was the TKI used in most of these reports and showed significant PFS in some, but not all cases^9^. There remains considerable uncertainty regarding optimal frontline therapy for advanced and metastatic NSCLC with *rare ALK* fusions.

This study aimed to compare the clinical characteristics, prognosis, and TKI efficacy in patients with *rare ALK* fusions to those with EML4-ALK fusions using a large international real-world dataset.

## Results

### ALK fusion prevalence and patient characteristics

In an international, retrospective, multicenter approach, we collected data from 26,152 NSCLC patients who underwent local NGS-testing capable of detecting both EML4-ALK and *rare ALK* fusions. An EML4-ALK fusion was identified in 509 (1.9%) and a *rare ALK* fusion in 55 (0.2%) patients. A total of 277 EML4-ALK and 51 *rare ALK* patients met the eligibility criteria and had sufficient clinical data for this analysis (see Supplementary Fig. 1 for details on patient disposition). First-line treatment for the advanced stage was started between 2013 and 2024.

Compared to the EML4-ALK cohort, patients with *rare ALK* fusions tended to be older and were more likely to be current or former smokers. Squamous histology was infrequently reported in the EML4-ALK cohort but occurred in 10% of the *rare ALK* patients (see Table 1 for full patient characteristics).

**Table 1:** Patient characteristics

Characteristics	EML4-ALK	*Rare ALK*
Total number of patients	**277**	**51**
Age, y, median (25th; 75th percentile)	59 (49; 69)*	66 (53; 72)*
Sex (*N* = 277, 51), *N* (%)		
Female	**143 (52)**	**21 (41)**
Male	**134 (48)**	**30 (59)**
Smoking (*N* = 266, 49), *N* (%)		
Active smoker	**32 (12)**	**11 (22)**
Former smoker	**63 (23)**	**19 (37)**
Never smoker	161 (58)*	19 (37)*
Pack years in smokers (*N* = 249, 48), mean	6.7*	14.7*
CNS metastases (*N* = 276, 51), *N* (%)		
CNS metastases at first diagnosis	**NA**	**11 (22)**
CNS metastasis at any time point	**104 (38)**	**19 (37)**
Histology at first diagnosis (*N* = 277, 51), *N* (%)		
Adenocarcinoma	**261 (94)**	**45 (88)**
Squamous-cell carcinoma	**4 (1)**	**5 (10)**
Adenosquamous carcinoma	**4 (1)**	**1 (2)**
Undifferentiated carcinoma NOS	**5 (2)**	
First line treatment in advanced stage (*N* = 264, 51), *N* (%)		
Crizotinib	**30 (11)**	**5 (10)**
Brigatinib	**50 (19)**	**2 (4)**
Ceritinib	**3 (1)**	**0 (0)**
Alectinib	**149 (56)**	**24 (47)**
Lorlatinib	**22 (8)**	**5 (10)**
Platinum-based chemotherapy	**0**	**7 (14)**
Other therapy	**0 (0)**	**3 (6)**
No therapy	**10 (4)**	**5 (10)**

In case of missing data, the number of individuals with available data is indicated next to the respective variable. Where data were completely available, no indication was made. Other therapies included: pembrolizumab (*N* = 1), capecitabin + temozolomide (*N* = 1), erlotinib + onartuzumab (*N* = 1).

*CNS* Central nervous system, *NA* not available.

*Difference statistically significant by t-test or Pearson Chi-square at alpha 0.05.

We detected a total of 21 different fusion partners, of which 15 occurred only once. The most common fusions were KIF5B-ALK (24%), HIP1-ALK (12%), GCC2-ALK (10%), and KLC1-ALK (10%). In all *rare* ALK fusions reported, ALK was the 3’ fusion partner preserving an intact tyrosine kinase domain (exons 20 to 29). A complete list of fusions with breakpoints is provided in Supplementary Table 1.

**Fig. 1:**
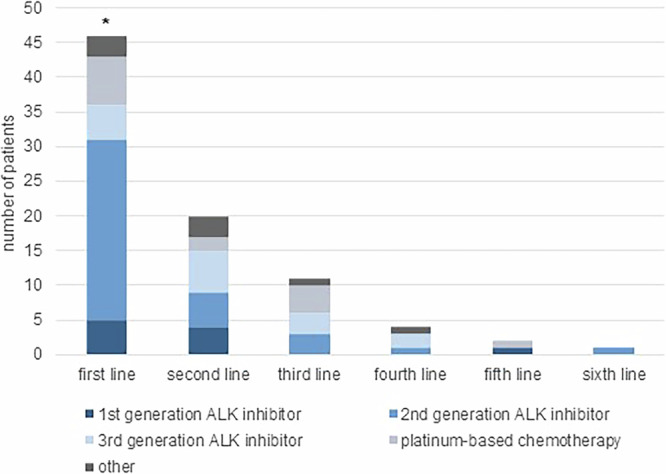
Treatment strategy per line of palliative treatment in patients with *rare ALK* fusions and advanced NSCLC. *: 5 patients did not receive any antineoplastic treatment. *Other:* 1st line: 1x Pembrolizumab, 1x Capecitabine/Temozolomide, 1x investigational medication, 2nd line: 1x Docetaxel/Ramucirumab, 1x Pembrolizumab, 3rd line: 1x Docetaxel, 4th line: 1x Docetaxel/Ramucirumab.

TKIs, predominantly alectinib, were used as first-line treatment for advanced disease in nearly all patients with EML4-ALK (96%) but only in 71% of patients with *rare ALK*. In the latter group, 14% of patients received platinum-based chemotherapy and 10% best supportive care as the primary treatment approach. In contrast, none of the patients with EML4-ALK were managed with chemotherapy in the first-line setting, and only 4% were deemed unsuitable for any tumor-directed treatment. Of note, 18% of the subjects in the *rare ALK* group never received a TKI during the course of their disease (Table 1 and Fig. 1).

### Treatment outcome

**Fig. 2:**
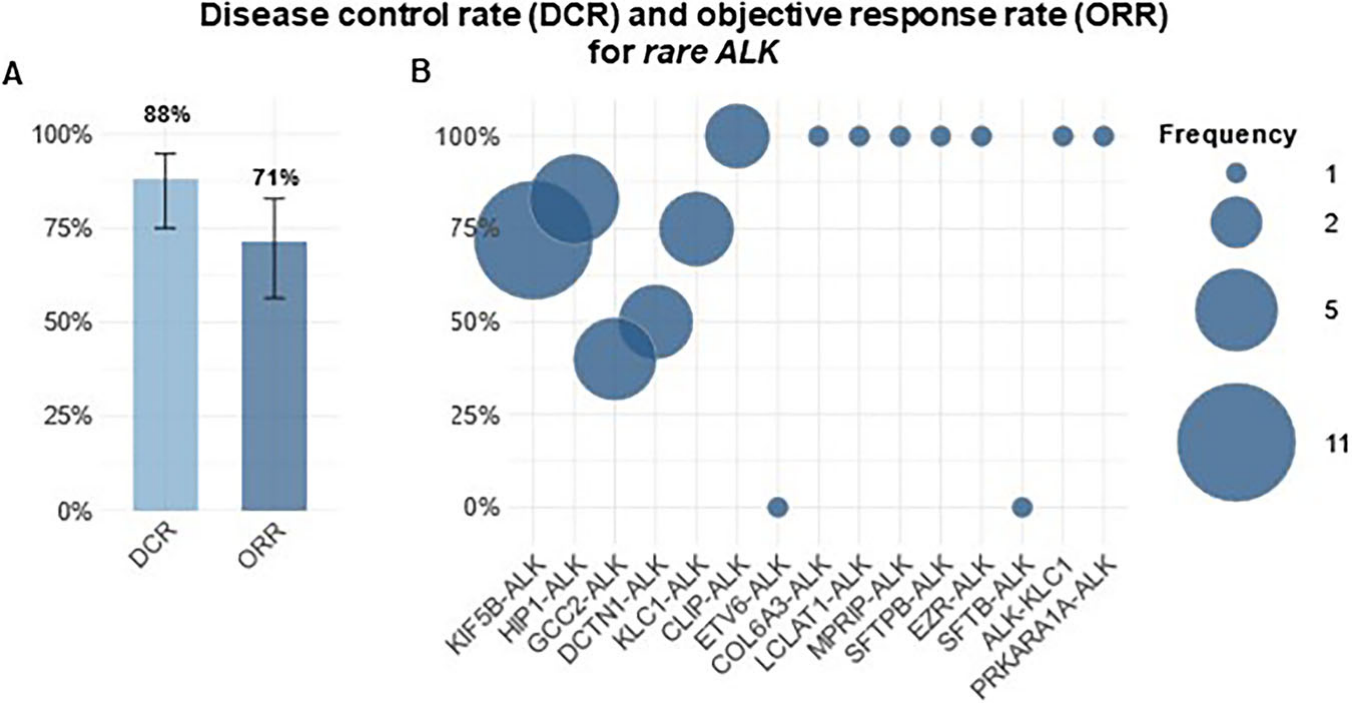
Disease control rate (DCR) and objective response rate (ORR) of the first ALK inhibitor in patients with *rare ALK* fusions. **A** DCR and ORR in patients with *rare ALK* fusions (*n* = 42) for first treatment with ALK TKI, regardless of treatment line. **B** ORR to first ALK TKI by fusion partner in patients with *rare ALK* fusions. The size of the bubble represents the number of patients in the subgroup, respectively. See also Supplementary Table 2 for presentation of the data in table format.

Overall survival for the entire cohort irrespective of treatment tended to be shorter in patients with *rare ALK* as opposed to EML4-ALK fusions (median 27 months vs. 57 months, HR 1.6, 95% CI 0.9–2.6, *p* = 0.08; Supplementary Fig. 2). However, when analyzing response to the first TKI ever received, efficacy of ALK inhibitors in patients with *rare ALK* was considerable with an objective response rate (ORR) of 71% (95% CI 56%–83%) and a disease control rate (DCR) of 88% (95% CI 73%–96%) (Fig. 2A).

Responses to TKI were observed in all types of *rare ALK* fusions (CLIP-ALK, DCTN1-ALK, GCC2-ALK, HIP1-ALK, KLC1-ALK) that occurred in multiple patients and in the majority (7/9) of fusions observed in only a single patient. Response rates ranged between 40% and 100% in the former group (Fig. 2B and Supplementary Table 2). In the latter group, the two non-responding patients (ETV6-ALK, SFTB-ALK) achieved prolonged disease stabilization beyond six months upon first exposure to a TKI. There was no evident association between the likelihood of response to a TKI and the specific exonic breakpoint at which the ALK fusion occurred (Supplementary Table 3). Among responders, all patients tested for ALK expression by immunohistochemistry (IHC) were IHC positive, while 3 out of 12 non-responders were IHC negative (Supplementary Table 4).

**Fig. 3:**
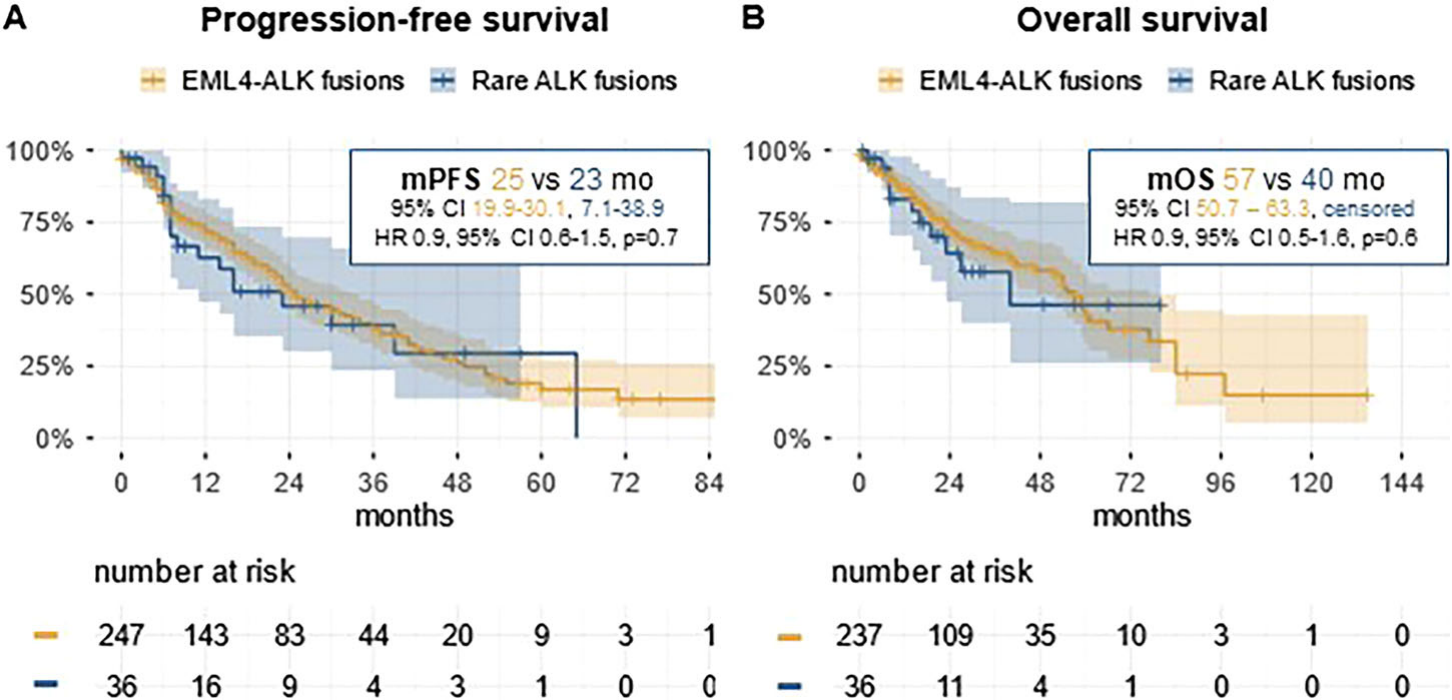
Efficacy of first-line TKI. Kaplan–Meier plots with 95% confidence intervals for **A** progression-free survival and **B** overall survival in patients with *rare ALK* fusions as compared to EML4-ALK fusions during first-line therapy with ALK inhibitors in advanced NSCLC.

When focusing on patients receiving an ALK inhibitor as first-line therapy, ORR was similar between EML4-ALK (85%, 95% CI 79–89) and *rare ALK* (74%, 95% CI 56–87; *p* = 0.9) groups. Median PFS was 25 months in the former and 23 months in the latter group (HR 0.9, 95% CI 0.6–1.5, *p* = 0.7) with 53% and 50% of PFS events having occurred, respectively (Fig. 3A). Overall survival (OS) was immature (maturity 34% and 31% of events having occurred in EML4-ALK and rare ALK, respectively). Nevertheless, survival curves were largely overlapping (median OS 57 vs. 40 months, HR 0.9, 96% CI 0.5–1.6, *p* = 0.6) for patients with EML4-ALK and *rare ALK* fusions receiving a TKI as first-line therapy (Fig. 3B).

**Fig. 4:**
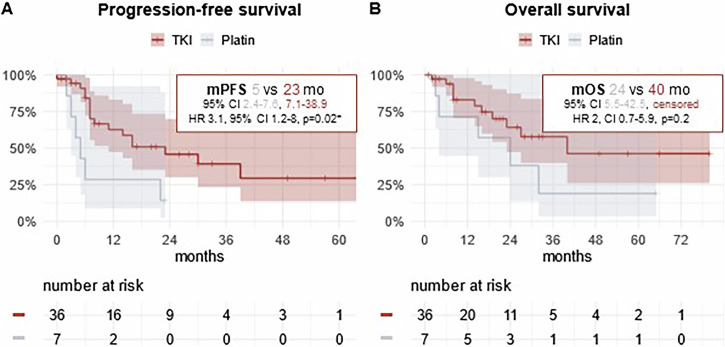
First-line chemotherapy vs. TKI in patients with *rare ALK* fusions. Kaplan–Meier-Curves with 95% confidence intervals for **A** progression-free survival and **B** overall survival in patients with *rare ALK* fusions to first-line therapy with platinum-based chemotherapy (gray) versus ALK TKI (red) in advanced NSCLC.

In contrast, the use of platinum-based chemotherapy as first-line treatment in advanced disease was associated with a significantly reduced PFS compared to treatment with TKI in patients with *rare ALK* (HR 3.1, 95% CI 1.2–8, *p* = 0.02, Fig. 4A). In addition, OS tended to be inferior in these patients (median 24 months vs. 40 months for platinum vs targeted therapy, HR 2, 95% CI 0.7–5.9) although this trend did not reach statistical significance (*p* = 0.2; Fig. 4B).

Beyond treatment with platinum-based chemotherapy, non-adenocarcinoma histology was also associated with shorter PFS in patients with *rare ALK* fusions (HR 9.6, 95% CI 2.6–36.3, *p* < 0.01). A history of smoking and an ECOG performance status of 2–3 showed a statistically nonsignificant trend towards impaired PFS (Supplementary Table 5). In the entire cohort patients, only use of TKI in first-line therapy (compared to chemotherapy) and age <65 years (compared to ≥65 years), but not the type of fusion (*rare ALK* vs. EML4-ALK) or other factors, were significantly associated with longer first-line PFS in multivariate Cox regression analysis (Supplementary Table 5).

## Discussion

The broad application of NGS gene fusion assays has led to an increasing number of *rare ALK* fusion partners being identified in NSCLC. The observed frequency of all ALK fusions (3.5% in adenocarcinoma, 1.9% in unselected NSCLC) in our cohort is in line with previous reports (2–8%)^10–12^, Among those, 11% had a fusion partner other than EML4 (*rare ALK*). Little is known about the specific efficacy of TKIs in this subgroup, with most of the evidence being derived from case reports or small case series6. In NSCLC, rare and atypical alterations may be relatively insensitive to standard targeted therapy, such as EGFR Exon 20 insertions^13^, or highly heterogeneous, like BRAF non-V600 mutations^14,15^.

In our real-world cohort, median OS was about 30 months shorter in *rare ALK* compared to EML4-ALK fusion positive patients (27 vs. 57 months). These differences may relate to tumor biology or patient characteristics, such as the higher proportion of current or former smokers and the greater median age observed in the *rare ALK* cohort. Another key difference between the two cohorts was the larger proportion of *rare ALK* patients receiving platinum-based chemotherapy or best-supportive care as frontline treatment, which was associated with inferior survival.

Among patients treated with a first-line TKI (mainly alectinib), ORR and PFS did not differ significantly between *rare ALK* and EML4-ALK patients (ORR 74% vs. 85%, median PFS 23 vs. 25 months), consistent with findings from two smaller Chinese single-center studies^16,17^. We observed clinical activity of ALK inhibitors across all fusion partners, which seems biologically plausible given that all fusions preserved the ALK tyrosine kinase domain. Even though the limited sample size and the absence of experimental evidence preclude a definitive conclusion, our data suggest that *rare ALK* fusions as a group should be seen as TKI-sensitive. Our findings also support first-line treatment with ALK-TKI in all ALK-positive patients, including those identified by ALK-IHC, which does not reveal the specific fusion partner.

Our study has several limitations inherent to its multicenter retrospective design. First, the exact estimation of fusion frequency is limited by heterogeneity in the NGS-assays and institutional testing strategy used across 29 different institutions. Response assessment did not undergo central review. The limited sample size restricted the ability to control for confounders. In addition, the differential outcome between TKI and chemotherapy-treated *rare ALK* patients might be due to selection bias, with patients presenting with an aggressive disease phenotype being more likely to receive chemotherapy upfront. The median PFS observed in our first-line TKI-treated *rare ALK* and EML4-ALK cohorts compares unfavorably to the alectinib arm of the phase III ALEX trial (23 and 25 months vs. 35 months). It is, however, similar to recently published real-world data^18^. Finally, we lack comprehensive data on the efficacy of third-generation ALK inhibitors since most patients were treated before lorlatinib became widely available and a standard-of-care first-line approach^19^.

In summary, our findings support the use of TKIs as first-line treatment in advanced NSCLC with *rare ALK* fusions.

## Methods

### Study design

We conducted a retrospective, multicenter chart analysis among 29 international lung cancer centers from 6 countries (Germany, Switzerland, Austria, Greece, Slovenia, Israel). Adult patients were eligible for inclusion if they met the following inclusion criteria: (1) histologically confirmed advanced-stage NSCLC without curative treatment options, (2) an ALK fusion detected by next-generation sequencing (NGS), and (3) a minimum follow-up of 3 months after initiation of first-line treatment. Patients with additional driver alterations were excluded. Data were collected for two cohorts: (a) the *rare ALK* cohort (defined as any ALK fusion with a partner other than EML4) for which a comprehensive dataset was compiled including patient characteristics, detailed NGS findings as reported by clinical routine testing, and clinical outcomes across all lines of therapy, and (b) a control cohort of patients with EML4-ALK fusions in which we collected a reduced data set focused on key patient characteristics and first-line targeted therapy. Detailed lists of variables collected for each cohort are provided below. Overall, the dataset was largely complete, though some variables (e.g., ECOG performance status, TP53 status) were occasionally missing at random.

List of variables. *Italic variables* are available in both cohorts, **bold variables** are available only in the rare ALK cohort. Patient characteristics: *Gender, Date of Birth, Smoking status, Pack years, Survival status, Date of death / last known alive, Date of 1st Diagnosis, UICC Stage at first diagnosis, Date of non-curative Stage III/IV diagnosis, Histology, CNS metastases at any time*. **CNS metastases at first diagnosis. ECOG performance status**. Molecular data: **Biopsy date, ALK IHC results, ALK FISH results, Full fusion gene specification (fusion gene 1** + **2 name, exonic breakpoint, RefSeq ID, Fusion cross reads), TP53 mutation, Other genetic findings, PD-L1 method, PD-L1 result**. Therapy data: *antineoplastic drug regimen* (**in EML4-ALK, only first-line**), *Start/Stop dates, Date of disease progression, best response, reason for discontinuation*.

In addition to individual patient data, we requested institution-level molecular testing data. Centers reported the total number of NSCLC patients (all histologic subtypes) tested with an NGS assay capable of detecting ALK fusions, and among those patients, the frequency of EML4-ALK and *rare ALK* fusions. Fusion detection was performed using NGS-RNA fusion panels, validated according to locally applicable legislation. We did not conduct central validation testing. The following panels have been used. **RNA-based panels**: *Archer® PanST Panel, Archer® FUSIONPlex® Lung v2, Oncomine™ Focus RNA Assay, Archer® FusionPlex® Lung v1, FusionPlex® CTL Panel, QIAseq® Targeted RNAscan Panel*. **Hybrid DNA/RNA panels**: *Oncomine™ Comprehensive Assay / nNGM v2 (v1.5), TruSight Oncology 500 (TSO500), Oncomine™ Precision Assay, Oncomine™ Focus Assay (Hybrid DNA* + *RNA), AmpliSeq™ for Illumina Focus Panel, Oncomine™ Dx Assay, Oncomine™ Dx Express (DNA + fusions), Oncomine™ Comprehensive Assay v3 Plus*.

### Analysis of patient characteristics and clinical endpoints

Our study aimed to compare the clinical characteristics of and treatment outcomes in patients with *rare ALK* to those with EML4-ALK. It was conducted as an exploratory study without a predefined statistical hypothesis. Predefined main efficacy endpoints were progression-free survival (PFS), overall survival (OS), and objective response rate (ORR).

Analyses were performed on available data. Missing data were not imputed. Patient characteristics were analyzed with appropriate descriptive statistics and compared with a t-test at alpha 0.05 or Pearson’s chi-squared test. Evaluation of treatment response was performed at the individual centers according to the Response Evaluation Criteria in Solid Tumors, version 1.1 (RECIST), assessed by the investigator or local radiologist without central review. 95% confidence intervals for ORR were calculated using the Wilson method.

PFS was analyzed using Kaplan-Meier estimates, calculated from the start of therapy to the date of progression, initiation of a new systemic anti-cancer therapy, or death. Data were censored at the date of last follow-up if none of these events had occurred at the time of analysis. OS was assessed using Kaplan-Meier estimates, calculated from the start of therapy until the date of death. For patients who were alive at their latest follow-up, data were censored accordingly. We performed Cox-regression analyses and estimated hazard ratios to compare survival outcomes.

Statistical analyses were performed with IBM SPSS Statistics (version 30.0) and R (R version 4.4.1, R Foundation for Statistical Computing). The figures and tables were generated using R and Microsoft Office 2019.

### Ethics approval and informed consent

The study was carried out in accordance with the Code of Ethics of the World Medical Association (Declaration of Helsinki) and was approved by the ethics committee of the Technische Universität Dresden, Germany (BO-EK-253062024). Informed consent was obtained if required by law.

## Supplementary information

Supplementary Information

## Data Availability

Informed consent of participating patients does not cover unrestricted publication of complete data. Upon request to the corresponding author, anonymized data can be made available if intended use is in compliance with informed consent.
