# Supplementary material for: Beyond EML4: efficacy of targeted therapy in lung cancer patients with rare ALK fusions – a real-world retrospective analysis
Source: NPJ Precis Oncol. 2026 Jun 29;10:248. doi: 10.1038/s41698-026-01486-y (PMC13314944; doi:10.1038/s41698-026-01486-y)
Supplement: Supplementary file 1 — Supplementary Information [file 41698_2026_1486_MOESM1_ESM.docx]

**SUPPLEMENTS**


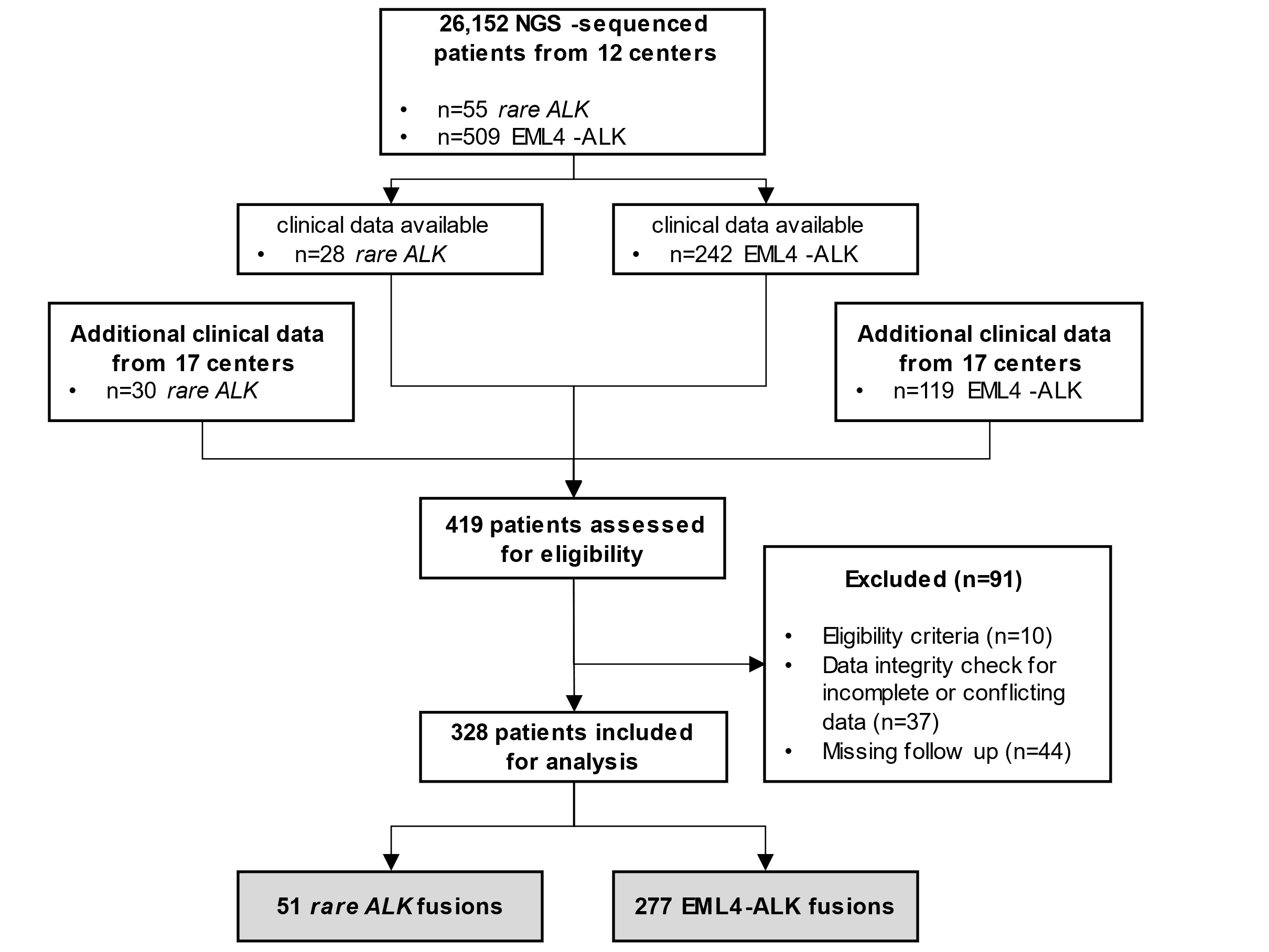


**Supplementary figure 1.** Study flowchart. Data was collected from 29 international centers and manually checked for eligibility, data quality and follow up (at least 3 months follow-up were required).


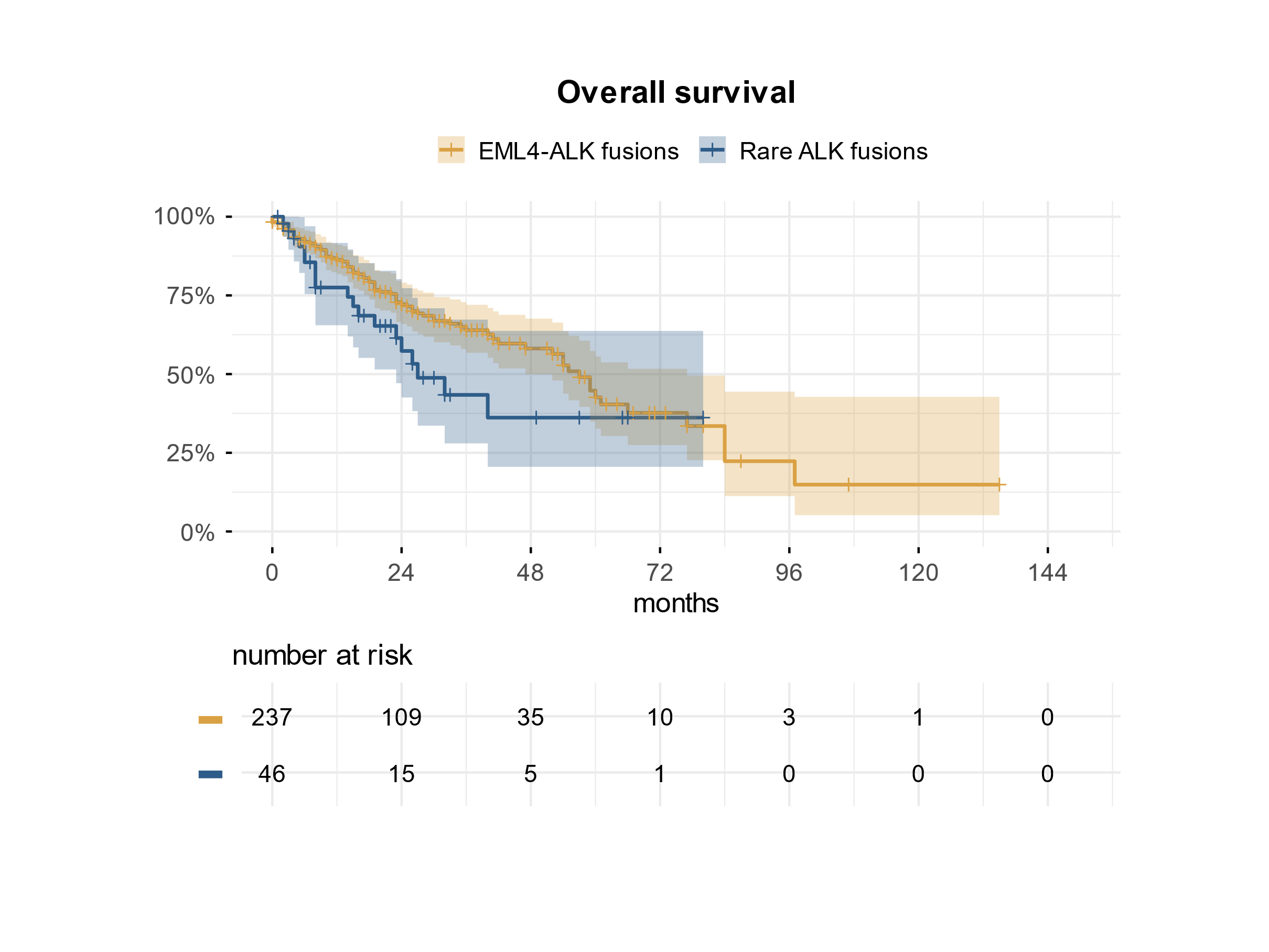


**Supplementary Figure 2.** Kaplan-Meier plot with 95% confidence intervals for overall survival in patients with *rare ALK* fusions as compared to EML4-ALK fusions during first-line therapy irrespective of treatment choice in advanced NSCLC.

| *Rare ALK* fusion variant | 5' fusion Exon | 3' fusion Exon | number |
| --- | --- | --- | --- |
| CLIP1-ALK | **13** | **20** | **1** |
| CLIP1-ALK | **12** | **20** | **2** |
| DCTN1-ALK | **5** | **2** | **1** |
| DCTN1-ALK | **26** | **20** | **3** |
| KIF5B-ALK | **NR** | **NR** | **1** |
| KIF5B-ALK | **15** | **20** | **1** |
| KIF5B-ALK | **24** | **20** | **5** |
| KIF5B-ALK | **17** | **20** | **5** |
| ALK-KLC1 | **20** | **9** | **1** |
| KLC1-ALK | **9** | **20** | **5** |
| HIP1-ALK | **21** | **20** | **3** |
| HIP1-ALK | **28** | **20** | **1** |
| HIP1-ALK | **30** | **20** | **1** |
| GCC2-ALK | **19** | **20** | **2** |
| GCC2-ALK | **13** | **20** | **1** |
| GCC2-ALK | **6** | **20** | **2** |
| ETV6-ALK | **NR** | **20** | **1** |
| KIF13A-ALK | **NR** | **20** | **1** |
| SDC-ALK | **10** | **1** | **1** |
| SFTB-ALK | **18** | **6** | **1** |
| COL6A3-ALK | **13** | **20** | **1** |
| LCLAT1-ALK | **1** | **2** | **1** |
| MPRIP-ALK | **NR** | **NR** | **1** |
| BCL11A-ALK | **3** | **20** | **1** |
| SFTPB-ALK | **6** | **18** | **1** |
| EZR-ALK | **12** | **18** | **1** |
| PRKAB1-ALK | **16** | **5** | **1** |
| CLIP4-ALK | **2** | **4** | **1** |
| FNDCA3-ALK | **19** | **20** | **1** |
| WDR43-ALK | **1** | **2** | **1** |
| PRKAR1A-ALK | **9** | **20** | **1** |

**Supplement Table 1.** Subgroups of *rare ALK* fusions sorted by fusion partner and exonic breakpoint.

| *Rare ALK* fusion partner | frequency | ORR (%) |
| --- | --- | --- |
| KIF5B-ALK | **11** | **72.2** |
| HIP1-ALK | **5** | **83.3** |
| GCC2-ALK | **5** | **40** |
| DCTN1-ALK | **4** | **50** |
| KLC1-ALK | **4** | **75** |
| CLIP-ALK | **3** | **100** |
| ETV6-ALK | **1** | **0** |
| COL6A3-ALK | **1** | **100** |
| LCLAT1-ALK | **1** | **100** |
| MPRIP-ALK | **1** | **100** |
| SFTPB-ALK | **1** | **100** |
| EZR-ALK | **1** | **100** |
| SFTB-ALK | **1** | **0** |
| ALK-KLC1 | **1** | **100** |
| PRKAR1A-ALK | **1** | **100** |

**Supplement Table 2.** Objective response rate to first treatment with ALK-TKI sorted by fusion partner.

| ALK fusion gene | 5' fusion partner | 3' fusion partner | ALK IHC | ALK FISH | 1st targeted treatment | Best response |
| --- | --- | --- | --- | --- | --- | --- |
| CLIP1-ALK | 12 | 20 | p | n | Lorlatinib | PR |
| DCTN1-ALK | 26 | 20 | p | n | Alectinib | PR |
| DCTN1-ALK | 26 | 20 | p |  | Crizotinib | SD |
| KIF5B-ALK | 17 | 20 | p | n | Alectinib | PD |
| KIF5B-ALK | 17 | 20 | p |  | Lorlatinib | SD |
| KLC1-ALK | 9 | 20 | p | n | Lorlatinib | PR |
| HIP1-ALK | 30 | 20 | p |  | Alectinib | PR |
| ETV6-ALK |  | 20 | n | p | Alectinib | SD |
| KLC1-ALK | 9 | 20 | p |  | Alectinib | PR |
| GCC2-ALK | 6 | 20 | p |  | Alectinib | PD |
| KIF5B-ALK | 17 | 20 | p |  | Lorlatinib | PR |
| HIP1-ALK | 21 | 20 | p |  | Brigatinib | PR |
| SFTB-ALK | 18 | 6 | n |  | Crizotinib | SD |
| KIF5B-ALK | 24 | 20 | p |  | Crizotinib | PR |
| KLC1-ALK | 9 | 20 | p | p | Crizotinib | SD |
| KIF5B-ALK | 24 | 20 |  |  | Crizotinib | PR |
| HIP1-ALK | 21 | 20 |  |  | Alectinib | PR |
| HIP1-ALK | 28 | 20 | p |  | Alectinib | SD |
| COL6A3-ALK | 13 | 20 |  |  | Crizotinib | PR |
| LCLAT1-ALK | 1 | 2 |  |  | Alectinib | PR |
| KIF5B-ALK | 17 | 20 |  |  | Alectinib | PR |
| KIF5B-ALK | 24 | 20 | p |  | Alectinib | PR |
| KIF5B-ALK | 17 | 20 | p |  | Alectinib | PR |
| GCC2-ALK | 19 | 20 | p |  | Alectinib | PR |
| KLC1-ALK | 9 | 20 | p |  | Alectinib | PR |
| DCTN1-ALK | 26 | 20 | p |  | Brigatinib | PR |
| HIP1-ALK | 30 | 20 |  | p | Brigatinib | PR |
| MPRIP-ALK |  |  | p | p | Alectinib | PR |
| KIF5B-ALK |  |  | p | p | Crizotinib | SD |
| KIF5B-ALK | 15 | 20 |  |  | Alectinib | PR |
| GCC2-ALK | 13 | 20 | p |  | Alectinib | PD |
| ALK-KLC1 | 20 | 9 |  |  | Alectinib | PR |
| SFTPB-ALK | 6 | 18 |  |  | Crizotinib | PR |
| HIP1-ALK | 21 | 20 |  |  | Alectinib | PR |
| CLIP1-ALK | 13 | 20 | p |  | Alectinib | CR |
| KIF5B-ALK | 24 | 20 |  |  | Alectinib | PR |
| EZR-ALK | 12 | 18 | p | p | Alectinib | PR |
| DCTN1-ALK | 5 | 2 | p | p | Alectinib | PD |
| GCC2-ALK | 6 | 20 | n | p | Alectinib | PD |
| PRKAR1A-ALK | 9 | 20 | p |  | Alectinib | PR |
| GCC2-ALK | 19 | 20 | p |  | Alectinib | PR |
| CLIP1-ALK | 12 | 20 | p |  | Lorlatinib | PR |

**Supplementary table 2.** *Rare ALK* cohort with information on fusion partner, break points, IHC and FISH. Best responses to first treatment with TKI, irrespective of treatment line. ORR data for first treatment with TKI available for 42/51 *rare ALK* patients (CR 1/42, PR 29/42, SD 7/42, PD 5/42). p: positive, n: negative.

|  | **Responder (N = 30)** | **Non-responder (N = 12)** |
| --- | --- | --- |
| **IHC positive** | **19** | **9** |
| **IHC negative** | **0** | **3** |
| **IHC not available** | **11** | **0** |
| **IHC positivity  in tested patients** | **100%** | **75%** |

**Supplementary table 3.** ALK expression by immunohistochemistry (IHC) in patients with *rare ALK* fusions with or without objective tumor response to first ALK inhibitor. FISH: fluorescence in situ hybridization.

| Variable | Availability of Data | Median PFS (95% CI) | Proportional hazards assumption | Hazard Ratio (95% CI) | P-value for Cox regression |
| --- | --- | --- | --- | --- | --- |
| Sex (female vs male) | **N = 51/51 (100%)** | **11 (0-23.6) vs 22 (0-24.8) mo** | **Not True** | **/** | **/** |
| Age (>65 years vs ≤65 years) | **N = 51/51 (100%)** | **14 (6.2-21.8) vs 30 (0-62.5) mo** | **Not True** | **/** | **/** |
| ECOG (PS 2-3 vs PS 0-1) | **N = 49 / 51 (96%)** | **8 (5.1-10.9) vs 16 (0-32.5) mo** | **True** | **2.1 (0.4-2.4)** | **0.1** |
| Histology (Other vs Adeno) | **N = 51/51 (100%)** | **3 (2.2-3.8) vs 16 (2.4-29.6) mo** | **True** | **9.6 (2.6-36.3)** | **<0.01** |
| Smoker (never vs ever) | **N = 49 / 51 (96%)** | **23 (2.4-43.6) vs 8 (0.2-15.8) mo** | **True** | **0.6 (0.3-1.4)** | **0.3** |
| Brain metastasis at first diagnosis (yes vs no) | **N = 49 / 51 (96%)** | **14 (7.0-21) vs 16 (0-37) mo** | **Not True** | **/** | **/** |
| TP53 Mutation (yes vs no) | **N = 43 / 51 (84%)** | **23 (6.3-30.7) vs 8 (2-14) mo** | **Borderline** | **/** | **/** |
| First-line Therapy (Platin vs TKI) | **N = 51 / 51 (100%)** | **5 (1.3-2.4) vs 23 (7.1-38.9) mo** | **True** | **3.1 (1.2-8)** | **0.02** |

**Supplementary table 4.** Univariate Cox regression analysis for first-line PFS in patients with *rare ALK* fusions.

| Step | Variable | Hazard Ratio (95% CI) | P-value for Cox regression |
| --- | --- | --- | --- |
| 1 | **EML4-ALK / rare ALK** | **1 (0.6-1.6)** | **0.9** |
|  | **Sex (female vs male)** | **1.1 (0.8-1.6)** | **0.5** |
|  | **Age (>65 years vs ≤65 years)** | **1.6 (1.2-2.3)** | **0.004** |
|  | **Histology (Other vs Adeno)** | **0.6 (0.3-1.2)** | **0.13** |
|  | **Smoker (never vs ever)** | **1 (0.7-1.4)** | **0.8** |
|  | **First-line Therapy (Platin vs TKI)** | **3.6 (1.5-8.5)** | **0.004** |
| 2 | **Sex (female vs male)** | **1.1 (0.8-1.5)** | **0.5** |
|  | **Age (>65 years vs ≤65 years)** | **1.6 (1.2-2.3)** | **0.004** |
|  | **Histology (Other vs Adeno)** | **0.6 (0.3-1.2)** | **0.13** |
|  | **Smoker (never vs ever)** | **1 (0.7-1.4)** | **0.8** |
|  | **First-line Therapy (Platin vs TKI)** | **3.5 (1.7-7.4)** | **0.001** |
| 3 | **Age (>65 years vs ≤65 years)** | **1.6 (1.1-2.3)** | **0.004** |
|  | **Histology (Other vs Adeno)** | **0.6 (0.3-1.2)** | **0.12** |
|  | **Sex (female vs male)** | **1.1 (0.8-1.5)** | **0.5** |
|  | **First-line Therapy (Platin vs TKI)** | **3.5 (1.7-7.2)** | **<0.001** |
| 4 | **Histology (Other vs Adeno)** | **0.6 (0.3-1.2)** | **0.13** |
|  | **Age (>65 years vs ≤65 years)** | **1.6 (1.2-2.2)** | **0.004** |
|  | **First-line Therapy (Platin vs TKI)** | **3.6 (1.7-2.4)** | **<0.001** |
| 5 | **Age (>65 years vs ≤65 years)** | **1.6 (1.1-2.2)** | **0.005** |
|  | **First-line Therapy (Platin vs TKI)** | **3.7 (1.8-7.7)** | **<0.001** |

**Supplementary table 5.** Multivariate Cox regression analysis on predictive factors for first-line PFS in the entire patient cohort. When comparing first-line PFS for patients with EML4::ALK and rare ALK fusions, we found a non-significant trend for inferior outcome for rare ALK fusions (median PFS 25 vs 14 months, HR 1.452, p=0.079). In order to explore, whether this trend might have been biased by clinical covariates, we generated a multivariate Cox regression model with all available potential confounders and performed step-wise exclusion of non-associated variates based on likelihood ratio (backwards LR approach). The variables included were smoking status (never vs ever), histology (adenocarcinoma vs other), age (≥65 vs <65 years), gender (female vs male), first-line therapy (TKI vs other), and ALK fusion (EML4-ALK vs rare ALK). The only variables that remained significantly associated with PFS in the final model were choice of first-line treatment (TKI vs chemo: HR 3.7 at p<0.001 in favor of TKI) and age (HR 1.6 at p=0.005 in favor of the younger group), but not the type of ALK fusion.
